# Prospective Comparative Study Between Subarachnoid Chloroprocaine and General Anesthesia for Short Daycare Surgeries

**DOI:** 10.7759/cureus.78973

**Published:** 2025-02-13

**Authors:** Manju, Bhavna Agarwal, Usha Choudhary, Mukesh Choudhary, Yogendra D Singh, Vijay P Nehra

**Affiliations:** 1 Anesthesiology, University College of Medical Sciences, Delhi, IND; 2 Anesthesiology, Mahatama Gandhi Medical College, Jaipur, IND; 3 Anesthesiology, Government Medical College, Pali, IND; 4 Anesthesiology, Government Medical College, Barmer, IND; 5 Anesthesiology, District Hospital, Kekri, IND; 6 Anesthesiology, Government Community Health Centre, Sikar, IND

**Keywords:** chloroprocaine, day care surgery, early postoperative discharge, general anesthesia, spinal anesthesia

## Abstract

Introduction: Daycare surgeries are increasingly possible due to advancements in anesthetic techniques, the availability of newer drugs, and newer surgical techniques. The anesthetic technique has to be modified and titrated to a level so as to provide optimal anesthesia with minimal side effects that will enable the patients to resume their daily activities. General anesthesia and newer airway adjuncts have completely changed non-invasive airway management in daycare surgeries. Chloroprocaine (CP) is an amino-ester local anesthetic with a very short half-life and is the recommended local anesthetic agent for spinal anesthesia in patients undergoing ambulatory surgeries because of its low incidences of adverse side effects, superior recovery profile, and raised operating room efficiency.

Method: A prospective, comparative, randomized, double-blinded study was performed on 60 patients. These patients were divided into two groups: Group C (CP) with 30 patients and Group G (general anesthesia) with 30 patients. The groups were compared for early postoperative recovery and discharge.

Result: The mean time of the first analgesic (min) was lower in Group G (15.2±3.0) than in Group C (26.3±3.04), and the difference was statistically significant. In our study, the maximum number of patients in Group C had a score of 12 compared to Group G, with a highly significant difference of 0.001, denoting that patients in Group C had more stable hemodynamic and ventilatory parameters and were ready to be discharged earlier than Group G patients. The mean time of discharge from PACU (min) was higher in Group G (49.5±6.4) than in Group C (22.7±4.5), and the difference was statistically significant.

Conclusion: Spinal anesthesia with CP is significantly superior to general anesthesia because patients who receive it have a longer initial rescue analgesic requirement duration, stable ventilatory and hemodynamic parameters, and an early mean time of PACU discharge.

## Introduction

Daycare surgery, also known as same-day surgery, or ambulatory surgery, is for patients who need a surgical procedure with minimal or no overnight admission to the hospital. These surgeries are accelerating due to advancements in anesthesia techniques and the availability of newer drugs. The anesthetic technique has to be modified and titrated to a level so as to provide optimal anesthesia with minimal side effects and the cost-effectiveness that will enable the patients to carry out their daily activities and return to normal life as soon as possible. Different techniques are used to deliver anesthesia to patients to ensure a fast and safe recovery. General anesthesia with fast-acting drugs provides a fast recovery that facilitates an early discharge [1]. General anesthesia and newer airway adjuncts, such as the ProSeal laryngeal mask airway, Combitube, and others, have revolutionized noninvasive airway management in daycare surgeries. Sevoflurane and desflurane have maintained their place in daycare surgery practice but are not cost-effective. Rocuronium, succinylcholine, atracurium, and cisatracurium remain the muscle relaxants of choice for daycare surgeries [2,3].

Spinal anesthesia is a safe and reliable technique for ambulatory surgeries of the lower abdomen and lower limbs [4,5]. Lidocaine is a short-acting local anesthetic agent and it is an agent of choice for outpatient procedures, but its intrathecal administration has been associated with transient neurological symptoms (TNS) [6,7]. Chloroprocaine (CP) is an amino-ester local anesthetic agent with a very short half-life [1]. CP is the recommended local anesthetic agent for spinal anesthesia in patients undergoing ambulatory surgeries lasting 45-60 min because of its low incidences of adverse side effects, superior recovery profile, and raised operating room efficiency. The hypothesis for this study was that the discharge time was shorter and the quality of recovery was better if the patient received a subarachnoid block with CP compared with general anesthesia for short daycare surgeries.

## Materials and methods

This was a prospective, comparative, randomized, double-blind study conducted in the Department of Anesthesia and Critical Care at Government Medical College and Associated Hospital, Kota, from June 2020 to October 2020. The study was approved by the hospital’s Ethics Committee, and written informed consent was obtained.

Sample size calculation

The sample size was calculated using computer software version 11.5.0.0. (MedCalc Software BVBA, Acacialaan 22, 8400 Ostend, Belgium). Based on a minimum mean difference of 25% in parameters with α=0.01 and β=0.20, the sample size for each group was estimated at 30, which was required to detect a significant difference between the groups.

Inclusion criteria

The inclusion criteria included adults of either sex, aged 18-60 years, with ASA Grades I and II, and Mallampati I and II; patients undergoing elective daycare surgeries lasting 45-60 min, in whom the risk of urinary retention is minimal.

Exclusion criteria

The exclusion criteria included patients with ASA Grades III and IV; those who refused participation; individuals with a history of significant neurological, psychiatric, neuromuscular, cardiovascular, pulmonary, renal, or hepatic disease; patients with known hypersensitivity to drugs; individuals with coagulopathy; those with infection or ulceration at the site of subarachnoid block; patients undergoing surgeries known to cause urinary retention or requiring catheterization; and those whose surgery time exceeded the usual duration due to procedural delays or any other cause.

Study design

Blinding was done using the "closed envelope method," and the patients were divided into two groups of 30 each. Group C (n=30): Patients were administered 3 mL of 1% CP for subarachnoid block (30 mg). Group G (n=30): Patients were taken under general anesthesia with short-acting drugs in the usual way, with supraglottic devices like LMA and I-gel.

A detailed pre-anesthetic evaluation and routine investigations (hematological, fasting and random blood sugar, blood urea, serum creatinine, chest X-ray, and ECG) were performed. All patients were called on the morning of the surgery and informed about the anesthetic technique. Baseline vitals were recorded, and the intravenous line was secured. Patients were given 15 mL/kg RL as preloading, and monitors were attached according to ASA guidelines.

Group C

Spinal anesthesia was performed under aseptic conditions using a 25G Quincke’s spinal needle in a sitting position, with 3 mL of 1% CP. The onset and degree of the sensory blockade, onset and degree of motor blockade, duration of analgesia, quality of analgesia, recovery of the sensory block, duration of motor block, and quality of operative conditions were assessed.

Group G

Injections of glycopyrrolate (4 mcg/kg IV) and fentanyl (1.5 mcg/kg IV) were given. The induction of anesthesia was achieved by the inducing agent propofol (2 mg/kg IV). The airway was secured with an appropriate-sized LMA or I-gel. In cases where a muscle relaxant was required, an intermediate-acting non-depolarizing muscle relaxant, such as atracurium or cis-atracurium, was administered. Anesthesia was maintained with oxygen and a variable rate of sevoflurane. After surgery, residual neuromuscular blockade was reversed using neostigmine (0.05 mg/kg IV) and glycopyrrolate (0.01 mg/kg IV) if a muscle relaxant had been used. The LMA/I-gel was removed, and the patient was transferred to the postoperative recovery room.

Cardio-respiratory parameters of heart rate, blood pressure, and SpO_2_ were monitored continuously and recorded before (baseline) and every five minutes after subarachnoid block and general anesthesia performance until the end of surgery. All patients were observed for any complaints of pruritus, nausea, vomiting, respiratory depression, and post-spinal shivering that were carefully observed, recorded, and managed symptomatically. The pain intensity was determined by the Visual Analog Scale (VAS) for Pain. Oral tramadol was used for postoperative pain management.

The patients were interviewed after one day of surgery for their response to the whole procedure and were graded using a six-point scale for their satisfaction and acceptance level.

The surgeons were interviewed after one day of surgery. The response of the surgeon to the whole procedure was graded using a three-point scale for their satisfaction.

The readiness of discharge of patients from the ward was decided based on the fast-track eligibility criteria score, the patient’s post-operative recovery, and complications like urinary retention, postoperative nausea vomiting, giddiness, and muscle weakness.

Statistical evaluation

Statistical analysis was performed using Statistical Package for Social Sciences (SPSS) version 24.0 (IBM Corporation, Chicago, USA). Continuous variables were tested for normal distribution by the Kolmogorov-Smirnov test. A student's t-test was used for differences in hemodynamic variables between the groups and repeat measures of the ANOVA of intergroup evaluation. Nominal data was analyzed using the chi-square test or Fisher exact test. A p-value of <0.05 was considered statistically significant.

## Results

There was no significant difference in the demographic data, which included the patient’s age, sex, BMI, ASA grading, and duration of surgery and anesthesia between the two groups, as shown in Table 1.

**Table 1:** Demographic data along with duration of surgery and anesthesia

	Group C	Group G	P-value
Age (years)	34.45±8.77	37.36±7.84	0.716
Sex (M:F)	8/22	7/23	0.078
BMI (kg/m^3^)	20.6±1.7	21.3±1.3	0.104
ASA (I/ II)	24/6	25/5	0.138
Duration of surgery (min)	30.9± 5.1	31.1±5.9	0.086
Duration of anesthesia (min)	35.7±7.1	36.1±7.4	0.221

In Group C, the onset of sensory block was quick (3.3±0.7 min), with the highest level of sensory block at T8 occurring at 5.3±0.8 min. The duration of the sensory block was 57.4±3.3 min, the onset of the motor block was 4.6±0.8 min, and the duration of the motor block was 45.8±4.4 min, as shown in Table 2.

**Table 2:** Parameters assessed during anesthesia under chloroprocaine

Parameters	Mean (min)	SD
Onset of sensory block	3.3	0.7
Highest level of sensory block	5.3	0.8
Duration of sensory block	57.4	3.3
Onset of motor block	4.6	0.8
Duration of motor block	45.8	4.4

In Group C, the number of complete motor blockades was 19, the number of almost complete motor blockades was eight, and the number of partial motor blockades was three, as shown in Table 3.

**Table 3:** Degree of motor blockade in Group C (modified Bromage scale)

Degree	Number of patients
0	19
1	8
2	3

The mean time to first analgesia (min) was lower in Group G (15.2±3.0) than in Group C (26.3±3.04), and the difference was statistically significant, indicating that patients who received general anesthesia complained of pain earlier than those who received spinal chloroprocaine, as shown in Table 4.

**Table 4:** Comparison of the time of the first analgesia (min) between the groups

Groups	Mean (min)	SD	P-value
Group G	15.2	3.0	0.001
Group C	26.3	3.04	

In our study, the maximum number of patients in Group C had a score of 12 compared to Group G, with a highly significant difference of 0.001, denoting that patients in Group C had more stable hemodynamic and ventilatory parameters and were ready to be discharged earlier than Group G patients as seen in Table 5.

**Table 5:** Comparison of fast-track criteria for discharge of patients from PACU between the groups

Score	Group G (number of patients)	Group C (number of patients)	P-value
8	5	1	0.001
9	5	1	
10	3	1	
11	13	3	
12	4	24	

The mean time of discharge from PACU (min) was higher in Group G (49.5±6.4) than in Group C (22.7±4.5), and the difference was statistically significant as seen in Table 6.

**Table 6:** Comparison of time of discharge from PACU (min) between the groups

Groups	Mean (min)	SD	P-value
Group G	49.5	6.4	0.004
Group C	22.7	4.5	

The number of patients in Group G had more side effects than Group C, but the difference was statistically insignificant, denoting both groups were comparable as seen in Table 7.

**Table 7:** Distribution of side effects between the groups

Side effects	Group G (number of patients)	Group C (number of patients)
Hypotension	5	3
Bradycardia	2	1
Nausea	1	1
Vomiting	1	0
Itching	1	0
Neurological side effects	0	0
Total	10/30	5/30
P-value	0.13	

More patients in Group C had a grade 1 score compared to Group G, which had a significant difference, denoting patients in Group C were more satisfied and had fewer complaints as seen in Table 8.

**Table 8:** Patients’ satisfaction score (VAS score) VAS, Visual Analog Scale

Grade	Group G (number of patients)	Group C (number of patients)	P-value
Grade 1	22	28	0.037
Grade 2	5	2	0.227
Grade 3	3	0	-
Grade 4	0	0	-
Grade 5	0	0	-
Grade 6	0	0	-

More surgeons in Group C had a grade 1 score compared to Group G, but the difference was insignificant, denoting that both groups were comparable with respect to surgeon satisfaction as seen in Table 9.

**Table 9:** Surgeons’ satisfaction score

Grade	Group G (number of patients )	Group C (number of patients)	P-value
Grade 1	26	28	0.389
Grade 2	4	2	0.614
Grade 3	0	0	-

In Group G, 21 patients (70% of the patients) showed a score of 1, and in Group C, 28 (93.33% of the patients) patients showed a score of 1, with a statistically significant difference denoting that the readiness of discharge in Group C patients was better than Group G patients as seen in Table 10.

**Table 10:** Comparison of readiness for discharge between the groups

Groups	Score 1 (number of patients)	Score 2 (number of patients)
Group G	21	9
Group C	28	2
P-value	0.024	0.195

## Discussion

Comparison of discharge time from PACU

In this study, we used the fast-track eligibility score. The present study showed that the mean time of discharge from PACU was higher in Group G (49.5±6.4 min) than in Group C (22.7±4.5 min). This finding is similar to the study by Volker et al. (2018) [8], which showed that the mean time to discharge from the PACU was longer in Group G (142 min) than in Group C (117 min). Siddaiah et al. (2019) [9] demonstrated that the duration of discharge under CP was shorter (33.2±6.2 min) compared to the control group.

Comparison of first rescue analgesia time

The present study showed that the mean time of the first analgesic was significantly lower in Group G (15.2±3.0 min) than in Group C (26.3±3.04 min). Siddaiah et al. (2019) [9] showed that the mean time of the first analgesic under CP was much shorter in comparison to the control group.

Researchers conducted many studies to explore different doses of CP to confirm adequate efficacy and rapid resolution of block in outpatient procedures. Sell and Pitkanen et al. (2008) [10] examined four different doses of intrathecal CP (35, 40, 45, and 50 mg) in 64 patients posted for lower limb procedures. The sensory block regression and time to discharge were faster in the lower dose groups (35 and 40 mg). The higher level of block and time to complete block regression were comparable in all four groups.

Kopacz et al. (2005) [11] tested 10 and 20 mg of plain CP to obtain the minimum effective intrathecal dose. They found that 10 mg plain CP is not effective for spinal anesthesia. Similarly, the 20 mg dose did not provide dense motor block and sensory anesthesia to at least L1 in all subjects.

Casati et al. (2006) [12] tested three different intrathecal doses of CP (30, 40, and 50 mg) in 45 patients posted for elective lower limb surgeries of less than 60 min of duration with sensory anesthesia up to T10. They concluded that a 30 mg dose may not be suitable for lower limb procedures extending up to 60 min.

In our study, the highest level of sensory blockade achieved was T8 with the short-acting local anesthetic agent CP in spinal anesthesia. Hence, the time for rescue analgesia was minimal when compared to general anesthesia.

Comparison of postoperative complications in PACU

Our study showed that the number of patients (10 out of 30) who experienced side effects in Group G (five patients showed hypotension, two showed bradycardia, one showed nausea, one showed vomiting, and one showed itching) was greater than the number of patients (five out of 30) in Group C (three patients showed hypotension, one showed bradycardia, and one showed nausea). There was no significant difference between the groups with respect to the incidence of adverse effects. Sore throat after general anesthesia occurred in 17.5-23.9% of patients [13,14]; unspecific headaches were given an incidence of 10-17% after spinal anesthesia; and backache at the site of puncture was found in 7.5-14% of patients after uneventful spinal anesthesia [15,16].

AcStudies by L’Hermite et al. (2017) [14] and Siddaiah et al. (2019) [9] showed that patients under CP showed lesser side effects than the patients in the control group.

After spinal anesthesia with longer-acting local anesthetics, it is a prerequisite that the patient should void urine before discharge to avoid urinary retention (Kamphuis et al., 1998) [17]. Mulroy et al. (2009) [18] suggested relaxing the requirements for voiding before hospital discharge in outpatients receiving a spinal block with short-duration drugs and undergoing surgical procedures at low risk of urinary retention, such as lower limb surgery.

Besides urinary retention, another possible complaint after spinal anesthesia is the occurrence of TNS, for which the lithotomy or flexed knee positions are independent risk factors (Etezadi et al., 2013) [19]. In the retrospective analysis by Hejtmanek and Pollock et al. (2011) [20], no cases of TNS-like symptoms or neurotoxicity were reported, and the preservative-free formulation of 2-CP has become the short-acting local anesthetic of choice as a safe and effective alternative to lidocaine and procaine for short ambulatory procedures.

In our study, there was no incidence of TNS, which is consistent with the findings of Hejtmanek and Pollock et al. (2011) [20], possibly due to the new preservative-free formulations of CP available on the market. Since it was a daycare procedure, further follow-up during the postoperative period of up to seven days was necessary to detect evidence of TNS, which can be considered a limitation of our study.

Comparison of patients and surgeons satisfaction

Our study showed that patients’ satisfaction scores and surgeons' satisfaction scores were significantly higher in Group C in comparison to Group G. Volker et al. (2018) [8] reported that the majority, 98% of patients, were highly satisfied with their anesthesia. The difference between patient satisfaction and quality of recovery is crucial, as patients are usually very pleased with their anesthesia technique, as cited by Royse CF et al. (2013) [21]. Barnett SF (2013) [22] indicates that costs are affected by the quality of recovery, which can result in delayed discharge and increased nursing intensity.

Limitations of the study

Follow-up of patients was limited to discharge since it was a daycare surgery. Hence, delayed complications, such as neurological problems, post-dural puncture headaches, and back pain, may not have been observed. Sensory and motor blockade cannot be assessed under general anesthesia. The duration of surgery depends on the surgeon's experience and skills, and complications may occur intraoperatively. The use of analgesics, such as opioids and nonsteroidal anti-inflammatory drugs in general anesthesia, can interfere with the results of rescue analgesia. Complications like urinary retention, sore throat, and hypotension may increase the duration and cost of hospital stays.

Suggestions for further studies

Further follow-up of patients after discharge is needed to evaluate any delayed complications. Additional studies are required to improve the quality of anesthesia in daycare surgery by adding intrathecal adjuvants to preservative-free chloroprocaine. A larger sample size and a longer study duration, with prospective follow-up, could improve the validity and outcomes of the study.

## Conclusions

In summary, both methods of anesthesia for outpatient procedures have been found to be well-tolerated and reliable. Spinal anesthesia with CP is significantly superior to general anesthesia as it provides adequate duration and depth of surgical analgesia, early ambulation, discharge, and cost-effectiveness. The patient can tolerate liquids by mouth, walk unassisted, and manage nausea and pain with oral medications, leading to more rapid intermediate recovery. Because spinal anesthesia requires less postoperative pain and fewer analgesic requirements, promotes early recovery and ambulation, reduces postoperative nausea and vomiting, allows patients to resume early bowel and bladder activity, reduces the risk of postoperative respiratory depression and breathing difficulties, and eliminates the need for airway management, it is preferred over general anesthesia. Patients also report greater satisfaction and comfort with spinal anesthesia. One of the techniques used to anesthetize patients for short outpatient procedures across a wide range of surgeries is the plain CP subarachnoid block.
